# Operations for Otorrhœa

**Published:** 1908-03-14

**Authors:** 


### Otology.

### OPERATIONS FOR OTORRHŒA.

WITH ACUTE MASTOIDITIS—Part I.

Acute mastoiditis originates in the muco-periosteum lining the accessory cells of the tympanic cavity. In the majority of cases the disease is secondary to the middle ear suppuration: mastoiditis is rarely caused by disease of the external auditory canal, and is also rarely an expression of some systemic infection. In all cases of acute suppurative otitis media pus is liable to collect in the antrum and other accessory cells. This may be due to posture and imperfect antral drainage, or to the hyperæmia and hypersecretion with retention of mucus, from inflammatory obstruction in the upper tympanic spaces.

Clinically, we recognise cases belonging to two main classes: (1) those associated with recent acute otitis media; (2) others associated with chronic otorrhœa. Anatomical variations in the formation of the mastoid cells play an important rôle in determining the manifestations, course, diagnosis, and treatment of mastoiditis. These cells may be extensive or limited: they always communicate with one another and directly or indirectly with the antrum. It is estimated that the outer wall of the antrum is composed of compact non-cancellous bone in 46 per cent. of healthy individuals; that in nearly 25 per cent. numerous small cells open into the outer wall of the antrum; while in about 30 per cent. the outer cells are large and conspicuous. The apex of the mastoid is more frequently composed of cancellous bone without accessory cells, but the whole mastoid may be diffusely excavated by the development of accessory cells of every kind. The importance of a knowledge of these variations will be appreciated when we state that every mucous-lined cell must be completely extirpated in the operative treatment of mastoid suppuration.

Three distinctive morbid conditions of the contents of the mastoid antrum and cells are met with. (1) The cells may be full of pus, the lining membrane being intensely injected and inflamed, but not much swollen. (2) The antrum or cells may be occupied by vascular granulations, which represent swollen and proliferating muco-periosteum. (3) Besides pus, the antrum may contain yellow or brownish masses of cholesteatomatous material with a pearly-white covering. The central portion of the mass may be soft and putty-like. The walls of the cavity are generally remarkably smooth. Fœtor is common. Combinations of the above conditions are also found, more especially of (1) with (2) or (2) with (3).

In the bony walls of the antrum and cells there may be no obvious bone absorption: the diploe may be diffusely hyperæmic, especially in young subjects. There may be definite localised areas of softened bone, which can be excavated with small curettes, through the inner table, into the cranial cavity. A passage capable of admitting a probe may thus be formed. No such passages exist, or can be produced, in normal bone, by these means, with the small curettes. It is important not to overlook any of these infective pathways.

A portion of the wall of the sigmoid sinus may be completely absorbed, so that the sinus is in direct contact with the contents of the mastoid cavities. The roof of the antrum may exhibit similar changes, and the superior petrosal sinus may be infected by extension, while the lateral sinus is infected from the petrosal thrombus. Finally, the labyrinth may be involved.

With a view to serum therapy, it is important to recognise the specific organisms in mastoiditis. In primary acute otitis the pneumococcus has been most frequently found, while in acute mastoiditis following chronic suppurative otitis other streptococci are more common. Staphylococci are rarely found, except as the result of secondary infections.

The occurrence of general symptoms of malaise and pyrexia is the rule in mastoiditis. The temperature is, however, sometimes not markedly high, and is indeed occasionally normal. Pain and tenderness over the mastoid are the most characteristic features. The pain may be diffuse, but the tenderness is usually localised. The periosteum may be infiltrated, forming a swelling obliterating the post-auricular sulcus. The skin behind the pinna may be red, and pit on pressure. As the swelling increases between the mastoid and the concha, the latter is displaced away from the bone, causing the auricle to assume a peculiar position in a downward and outward direction. The otorrhœa, which may previously have been profuse, frequently decreases with the onset of acute mastoiditis.

Otoscopic examination often reveals a swelling of the roof and posterior wall of the external auditory canal. This swelling is deeper in the meatus than a furuncle. In early cases of acute otitis the mastoid symptoms may predominate, but the membrane may be found to be greatly inflamed or even bulging towards the meatus.

The inflammation may subside either spontaneously or after local applications and a brisk purge. Hot fomentations generally give relief, but sometimes the ice-bag or Leiter's tubes alleviate the pain more quickly. When a superficial mastoid abscess forms, it is apt to be followed by persistent mastoid fistula, unless treated by complete drainage of the mastoid cells. Occasionally the pus, instead of finding its way from the cells to the surface of the mastoid, perforates into the external auditory meatus, or it may track along the periosteal plane to the post-auricular groove. In cases of large apical mastoid cells, the pus may extend from these into the digastric fossa, leading to deep-seated cellulitis of the neck. Or the pus may spread direct into the sheath of the sterno-mastoid, causing acute suppurative myositis. And, lastly, the suppuration may invade the cranial cavity, giving rise to extra-dural abscess, infective sinus thrombosis, meningitis, or brain abscess.

